# Management of Reniform Nematode in Cotton Using Winter Crop Residue Amendments Under Greenhouse Conditions

**DOI:** 10.2478/jofnem-2023-0041

**Published:** 2023-10-21

**Authors:** Rebeca Sandoval-Ruiz, Zane J. Grabau

**Affiliations:** Entomology and Nematology Department, University of Florida, 1881 Natural Area Drive, Gainesville, FL 32611, United States

**Keywords:** *Avena sativa*, *Brassica carinata*, *B. napus*, biofumigation, canola, carinata, cotton, *Gossypium hirsutum*, hairy vetch, host, management, oat, reniform nematode, *Rotylenchulus reniformis*, *Vicia villosa*, winter crops

## Abstract

*Rotylenchulus reniformis* (reniform nematode, RN) is among the most important nematodes affecting cotton. Cultural practices, such as rotation and soil amendment, are established methods for managing RN. Management may be enhanced if crop residue has biofumigant properties against RN. The objective was to evaluate the efficacy of winter crop amendments for managing RN in the greenhouse. Reniform nematode-infested soil was amended with dry or fresh organic matter (OM, 2% w/w) from winter crops – canola, carinata, hairy vetch, oat, or no crop. Cotton was subsequently grown in this soil. Independent of the crop, dry OM amendments were more effective than no amendment at managing RN, while fresh OM amendments were not. Soil and root RN abundances and reproduction factors were generally lower in Trials 1 and 3 for dry OM than fresh OM amendments or control without OM. In Trial 2, none of the OM treatments reduced RN parameters compared with no OM control. In general, when compared to plants without RN or OM, RN did not produce significant changes in growth parameters but did affect physiology (Soil Plant Analysis Development, or SPAD, values). In conclusion, dry OM amendments can help manage RN, crop growth does not always relate to RN abundances, and SPAD values could help indicate RN presence.

Reniform nematode (*Rotylenchulus reniformis*, RN) is one of the most important pathogens in the southeast United States (Jones et al., 2013) and is considered one of the most significant plant-parasitic nematodes affecting cotton (*Gossypium hirsutum*) in the region (Robinson, 2007). Reniform nematode limits production, alters growth, and defers flowering, while the size and number of cotton bolls, as well as lint quality, are reduced (Koebernick et al., 2021). Yield losses caused by RN can reach 50% (Dyer et al., 2020).

Although there are relatively few practical chemical management options against nematodes in cotton production, nematicides are one of the most commonly used methods to control RN in cotton fields (Khanal et al., 2018). However, the proven negative effects of some nematicides on the environment and human health, as well as the relatively high costs of nematicides for producers (Riga, 2011) have led to a search for alternatives. Recently, reniform nematode-resistant cultivars have become commercially available, but grower adoption and evaluation of efficacy are still in relatively early stages (Turner et al., 2023). Therefore, cultural practices are critical in integrated RN management.

The application of organic matter amendments to soil, such as crop residues, is a cultural method that may improve soil properties and manage soil-dwelling pathogens, including nematodes (Oka, 2010). Application of organic amendments may suppress nematodes through various mechanisms (Oka, 2010) such as changing soil physicochemical conditions to impair nematode survival (Van Gundy, 1965; Oka and Yermiyahu, 2002), stimulating nematode antagonistic microorganisms (Rodriguez-Kabana et al., 1987), triggering plant tolerance and resistance to nematodes (Oka, 1999), or releasing compounds toxic to nematodes in a process often referred to as biofumigation (Kirkegaard et al., 1993). These toxic compounds involved in biofumigation are typically secondary metabolites produced by crops for defense against pests (Zaynab et al., 2019). Through these various mechanisms, soil amendments have the potential to be used for nematode management when crop residues are incorporated into the soil, either by terminating a rotation crop or introducing residue from a crop grown elsewhere (Stirling and Stirling, 2003; Cherr et al., 2006).

In the Southeast, some winter crop options are brassicas, cereals, and certain legumes. These crops vary in the amount and composition of biomass they produce (Sandoval-Ruiz and Grabau, 2023) as well as secondary metabolite production. Therefore, they may vary in their effectiveness at managing soil pathogens. Oat (*Avena sativa*) produces defense compounds called flavone-C-glycosides that inhibit nematode invasion and development (Soriano et al., 2004). Oat roots synthesize triterpene glycosides (avenacins), compounds that confer resistance to some soil pathogens (Mylona et al., 2008). The incorporation of mature dry tissues of oat has suppressed the incidence of *Meloidogyne* spp. (Akhtar and Alam, 1993), but this has not been previously studied in the region for RN. Hairy vetch (*Vicia villosa*) biosynthesizes cyanides (Kamo et al., 2006), which are produced after plant tissue maceration by enzymatic hydrolysis of cyanogenic glycosides by β-glucosidase enzymes (Poulton, 1990; Cressey and Reeve, 2019). Synthetic cyanamide had been used as a fertilizer with nematicidal effects (D’Addabbo et al., 1996). For instance, the application of hairy vetch dry organic matter at 0.5% w/w under greenhouse conditions reduced the reproduction of RN compared to the control without organic matter (Gardiano et al., 2013). Brassica plants have been widely used to manage some plant parasitic nematodes because they contain glucosinolates, which are substances that release biocidal isothiocyanates after being hydrolyzed by myrosinase enzymes (Halkier and Gershenzon, 2006; Vig et al., 2009). For example, it has been found that canola (*Brassica napus*) amendment applied as leaf leachate could suppress RN (Wang et al., 2001).

Another brassica of interest for nematode management in the Southeast is *Brassica carinata* (carinata). This emerging crop is produced for biofuel and has spurred commercial and research endeavors for its establishment in the region (Seepaul et al., 2019; Kumar et al., 2020). Carinata was recently described as a poor RN host, and was equally effective as oat, a common winter cover crop, at managing RN in greenhouse conditions (Sandoval-Ruiz and Grabau, 2023). However, the effects of carinata amendments on RN have not been studied.

In field conditions, winter cover crops can be incorporated into the soil as green organic matter (Treadwell, 2006). For instance, seed meals or green manure of brassica crops can be incorporated into the soil to manage nematodes (Dutta et al., 2019). In contrast, for carinata, dry residues would typically be incorporated into the soil after harvest so that the crop is allowed to fully mature and desiccate in the field before harvesting the seed for oil extraction. To our knowledge, there is no information about the influence of dry OM from carinata on RN. Therefore, evaluating the incorporation of both dry and fresh organic matter (OM) of winter crops is important for assessing the value of these crops to manage RN. This information is crucial for the establishment of nematode management practices.

The objectives of this research were to determine the effects of Southeast winter crops – canola, carinata, hairy vetch, and oat – on RN management and cotton growth and physiology when applied at 2% w/w as dry or fresh organic matter (OM) under greenhouse conditions.

## Materials and Methods

### Experimental design

This study was conducted in a repeated greenhouse pot experiment in a polycarbonate greenhouse at the University of Florida Entomology and Nematology Department in Gainesville, FL. The arrangement was a completely randomized design with six replicates and one factor: winter crop treatment. The experiment was performed three times (Trial 1, Trial 2, and Trial 3). A total of 10 treatments were evaluated: fresh shoot residue from each of the four winter crops (carinata, canola, oat, or hairy vetch) incorporated into soil independently (1–4); dry shoot residue from each of the winter crops incorporated into soil independently (5–8); and unamended control with RN (Ctrl) (9) or without RN (Ctrl−) (10) inoculation. Cultivars used were carinata “Avanza 641” (Nuseed Co., Sacramento, CA), the major carinata cultivar at the time this study was established; canola “Canterra 1918” (Canterra seeds, Manitoba, Canada); hairy vetch “Au merit” (provided by the University of Florida forage breeding program, Marianna, FL); and oat (unknown variety). Treatments were assessed in cotton cultivar “Deltapine 1646B2XF” (Bayer Crop Science, St. Louis, MO), as explained subsequently.

### Inoculum preparation

The RN population for this research came from a naturally infested field in Tift County, Georgia, and was maintained on cotton plants in greenhouse conditions. Inoculum was obtained based on methodology developed by Hussey and Barker (1973). Roots of cotton cultures were removed from soil, gently rinsed clean of the soil and cut into 2 cm pieces. Reniform nematodes (primarily eggs, but also vermiform nematodes) were then extracted from the roots by placing root sections in a 250-ml laboratory flask, covering roots with a 0.25% solution of sodium hypochlorite (NaOCl), and shaking for 1.5 min at 150 rpm in a VWR standard analog shaker 3500 STD (VWR International, PA, USA). The suspension was poured into 200-over-500-mesh sieves and washed for 30 seconds with tap water to remove the bleach. Reniform nematode eggs and vermiform stages that were retained on the 500-mesh sieve were collected in tap water for inoculum and quantified using a 400x PrimoVert (Carl Zeiss Inc., Thornwood, NY) inverted microscope. Nematodes were inoculated in each pot on the same day as inoculum extraction.

### Soil preparation

The soil used for the experiment was a Chipley-Foxworth-Albany complex (91% sand, 6.8% silt, and 2.4% clay with 1.7% OM) from the University of Florida North Florida Research and Education Center – Suwannee Valley, near Live Oak, FL. Soil was autoclaved at 121° C for 90 minutes in an Amsco Lab 250 LV autoclave (STERIS, Mentor, OH) before use in experiments.

### Trial establishment

Trial 1, Trial 2, and Trial 3 were established in November 2020, January 2021, and October 2021, respectively. More information about Trial schedules is provided in Table 1. This experiment had three stages: 1) winter crop growth, 2) organic matter incorporation and 3) evaluation. During the first stage, winter crops were grown for 74 to 76 days for the dry OM treatments and 82 days for the fresh OM treatments in 15 cm diameter clay pots containing 1000 cm^3^ (equivalent to 1500 g) of autoclaved soil that had not been inoculated with RN. Winter crops for the fresh and dry OM treatments were grown for different lengths of time to synchronize timing of OM incorporation for all treatments, since multiple days were needed to dry and grind the crop tissue for the dry OM treatments.

**Table 1.** Schedule for data collection and agronomic activities in greenhouse trials.

	**Trial 1**		**Trial 2**		**Trial 3**	

	**Date**	**DAP^a^**	**Date**	**DAP^a^**	**Date**	**DAP^a^**
1. Winter Crops						
1.1 Planting	08 November 2020	0	28 January 2021	0	18 October 2021	0
1.2 Harvest						
• Dry OM treatments	22 January 2021	75	12 April 2021	74	02 January 2022	76
• Fresh OM treatments	29 January 2021	82	20 April 2021	82	08 January 2022	82
1.3 Organic matter incorporation and nematode inoculation	30 January 2021	83	21 April 2021	83	09 January 2022	83
2. Cotton						
2.1 Planting	05 February 2021	0	26 April 2021	0	14 January 2022	0
2.2 Plant growth assessment	22 April 2021	76	11 July 2021	76	31 March 2022	76
2.2 Harvest	29 April 2021	83	17 July 2021	82	07 April 2022	83

a Days after planting

After the first stage, crop fresh shoots and roots were harvested from the original pots, so the residue could be used during the OM incorporation stage. Dry OM treatments were harvested, and the entire fresh plants were placed individually in paper bags in an oven (SPX Blue M, Electric, IL, USA) at 60° C for 7 to 9 days (Table 1). Subsequently, the dry tissue was ground in a domestic coffee grinder into sections of less than 0.5 cm. Afterward, the plants for fresh OM incorporation were harvested and entire plants (roots and shoots) were cut into pieces smaller than 0.5 cm. For both the dry and fresh OM treatments, the OM was homogenized in a plastic bag, and 30 g of fresh or 30 g of dry tissue was measured for incorporation into each pot (2% w/w for both dry and fresh OM treatments). This was 2% w/w for both fresh and dry OM treatments, the estimated amount of crop residues integrated into the soil in a commercial field (Stapleton and Duncan, 1998).

For each experimental unit, 1500 g of autoclaved soil, 6000 RN (eggs and vermiform RN), and the appropriate OM treatment were mixed in a plastic bag. Each mixture was placed in a new 15 cm diameter clay pot and left in the greenhouse for one week. During this period, pots were left uncovered to best simulate field conditions in cotton production, but were watered daily to maintain soil moisture. Subsequently, in each pot, four cotton seeds were planted and thinned to 2 plants per pot after 5 days. Cotton plants were then grown for 82 to 83 days until harvest. Plants were watered daily, and no fertilizer was added for any of the trials.

#### Nematode sampling and quantification

Reniform nematode abundances were assessed from the entire cotton root system and a subsample of soil at cotton harvest. Cotton plants and surrounding soil were removed from each pot and placed on a soil sifter with a metal mesh with 0.25 cm^2^ holes. Soil was dislodged from roots and screened through the mesh to homogenize it, and a subsample of 100 cm^3^ of soil was used for soil nematode extraction using the sucrose centrifugation method (Jenkins, 1964). Roots were separated from shoots by cutting, and subsequently gently washed with tap water and lightly dried on a paper towel. Then, nematode extractions from roots were done using the orbital shaker-sodium hypochlorite method (Hussey and Barker, 1973) as described previously. For RN root abundances, total RN population (eggs and vermiform nematodes) densities per gram of root and per root system were calculated. From soil, total RN were quantified per 100 cm^3^. Counting and identification were done using a 400X Carl Zeiss Primovert inverted microscope (New York, USA). The reproduction factor (RF) was then calculated (final population/initial population, where final population = RN eggs and vermiform in soil + roots) (Seinhorst, 1967).

#### Plant growth assessment

For cotton plants, plant height, Soil Plant Analysis Development (SPAD) chlorophyll analysis, and canopy cover were measured just before harvest. Root fresh weight, shoot fresh weight and shoot dry weight were measured just after harvest. Each plant variable was measured from two cotton plants per pot but, as pseudoreplicates, their values were averaged to report the final value for each replicate. Plant height was measured from the shoot base to the apical meristem. SPAD values were taken on the third fully-expanded leaf from the apex using a SPAD-502 chlorophyll meter (Konica Minolta Sensing Inc., Osaka, Japan). This technique has been broadly used in agriculture to provide a fast and non-destructive measure of the chlorophyll content in leaves (Ling et al., 2011). To assess canopy cover, each pot was placed in the center of a 1 m^2^ white piece of cardboard, and photos were taken at 1 m height, parallel to the top of the pot. Pictures were cut to only show 1 m^2^ using the computer imaging program GIMP (GNU Image Manipulation Program) 2.10.20 (The GIMP Team, Berkeley, CA) and were evaluated using Canopeo (Patrignani and Ochsner, 2015).

Fresh shoot weight was recorded immediately after harvest to avoid variations due to loss of water content. Cotton dry shoot weight was measured after drying plants in an oven in the method previously described for preparing dry OM treatments. Root weights were measured after separating them from the shoot, as described in the nematode quantification section.

#### Trial maintenance

The temperature was measured using a HOBO MX TidbiT 400 (Onset Computer Corporation, Bourne, MA). The average temperature while the winter crops were growing and incorporated was 18.3°C (max = 40.2°C, min = 6.0°C) for Trial 1, 20.1°C (max = 46.3°C, min = 9.2°C) for Trial 2, and 20.7°C (max=38.2°C, min = 10.0°C) for Trial 3. When cotton plants were growing, temperatures were 20.5°C (max = 42.1, min = 9.4°C) for Trial 1, 25.9°C (max = 42.3°C, min = 14.8°C) for Trial 2, and 21.6°C (max = 46.0, min = 6.3°C) for Trial 3. Plants were watered daily by hand and no supplemental light or fertilizer was used.

#### Statistical analysis

Data analysis was done in RStudio version 2021.09.0 (The R Foundation for Statistical Computing, Vienna, Austria). Normality was assessed by the Shapiro-Wilk test, and homogeneity of variances was measured by Levene's test. The nematode and plant parameters were analyzed separately because of trial-by-treatment interactions (ANOVA, *P* ≤ 0.05). The control without nematodes (Ctrl−) was not included when analyzing the nematode parameters but was included when the plant parameters were analyzed. The plant parameters that did not meet the statistical assumptions were ln(*x*+1) transformed to meet them. Plant parameters significantly affected by amendment treatments (ANOVA, *P* ≤ 0.05) were analyzed by Tukey's Honesty Significant Difference (HSD) test. The nematode variables did not meet the model assumptions after transformations, so nematode data were analyzed by Kruskal-Wallis non-parametric test. This was followed by multiple comparisons with Bonferroni's method at a 5% significance level, in instances where the Kruskal-Wallis's test was significant. In addition, a Spearman correlation between the nematode and plant parameters was done to determine whether, and how strongly, nematode and plant variables were associated.

In the figures, data is displayed as box plots. The black center line denotes the median (50^th^ percentile), the lower limit of boxes denotes the 25^th^ percentile, the upper limit of boxes denotes the 75^th^ percentile, whiskers indicate the 5^th^ and 95^th^ percentiles, and outliers represent values beyond the upper and lower whiskers, and are indicated as black dots.

## Results

### Reniform nematode soil abundances

**Figure 1:**
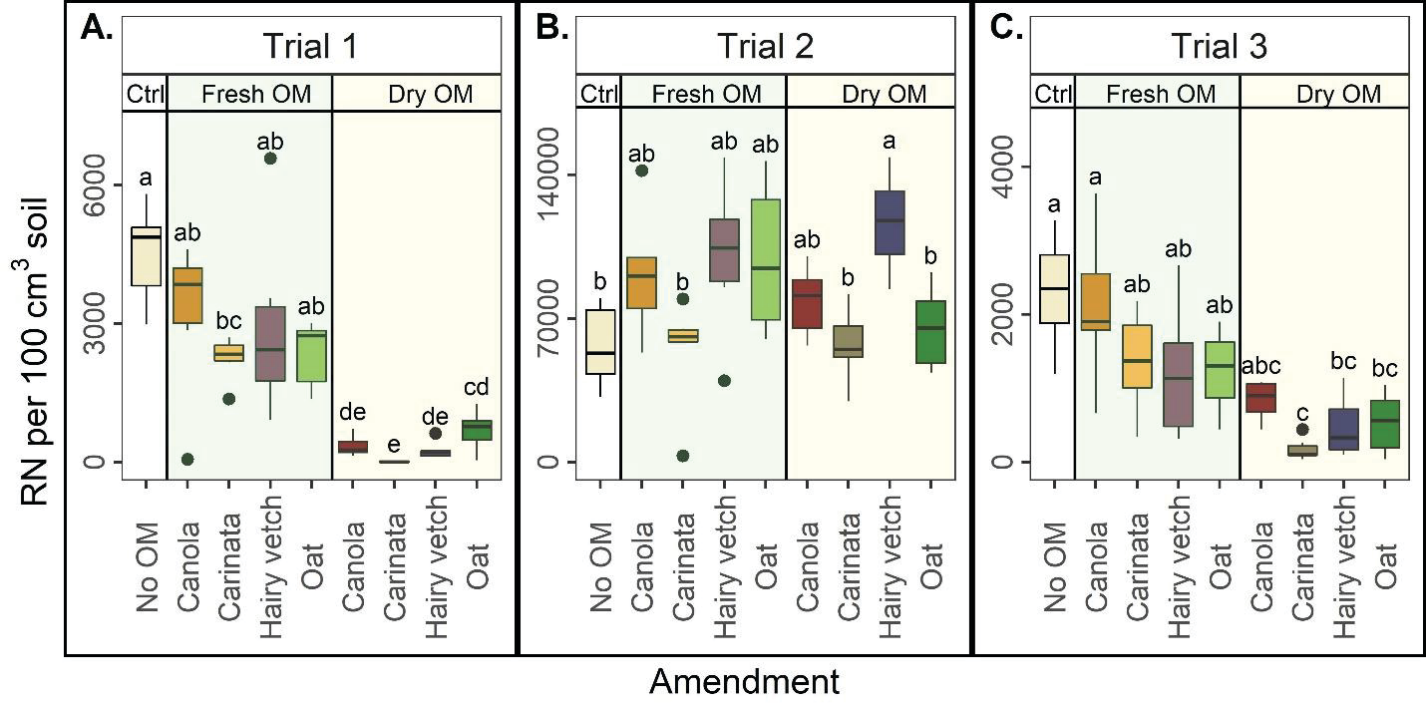
Reniform nematode (RN) in 100 cm^3^ of soil, by amendment, in Trial 1 (A), Trial 2 (B), and Trial 3 (C). Ctrl = control without organic matter (OM), Fresh OM= treatment with fresh OM, Dry OM = treatments with dry OM. Letters indicate treatment separation by Kruskal-Wallis with Bonferroni's method, *P* < 0.05. Data is displayed as boxplots.

Treatments significantly affected RN soil abundances in each trial, but trends varied by trial. In Trials 1 and 3, dry OM amendment was generally more effective than fresh OM, generally reducing RN soil abundances relative to the Ctrl for all winter crops (Fig. 1). In Trials 1 and 3, fresh OM, regardless of winter crop, generally did not reduce RN soil abundances compared to the Ctrl. In Trial 2, no treatments were effective, as RN soil abundances for all winter crops, regardless of OM type (fresh or dry), were similar to or greater than the Ctrl. Generally, there were minimal differences among winter crops within each OM amendment type (Fig. 1).

#### Reniform nematode root abundances

**Figure 2:**
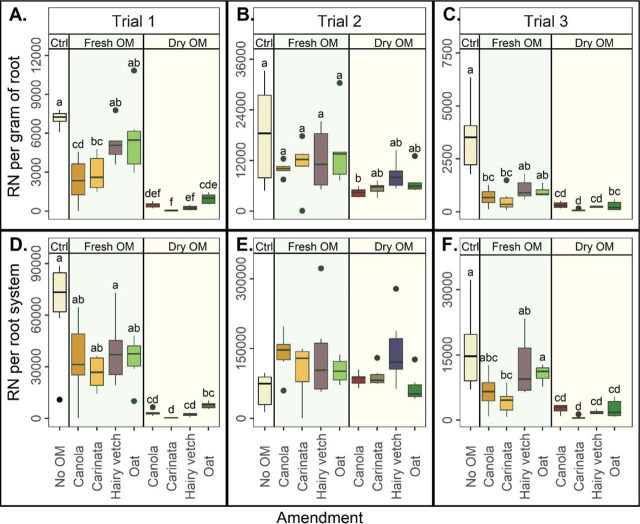
Reniform nematode (RN), by amendment, per gram of root in Trial 1 (A), Trial 2 (B), and Trial 3 (C) and in the root system in Trial 1 (D), Trial 2 (E), and Trial 3 (F) Ctrl = control without organic matter (OM), Fresh OM = treatment with fresh OM, Dry OM = treatments with dry OM. Letters indicate treatment separation by Kruskal-Wallis with Bonferroni's method, *P*<0.05. Data is displayed as boxplots.

There were significant main effects of amendments on RN/g root in all trials and on RN/root system in Trials 1 and 3 (Fig. 2). In general, the dry OM amendments were more effective at reducing RN abundances in the roots than the fresh OM amendments (Fig. 2). In Trials 1 and 3, dry OM amendments reduced the RN abundance per root system or gram of roots compared to the Ctrl. Carinata fresh OM significantly reduced RN/g root in Trials 1 and 3 and reduced RN/root system in Trial 3, both relative to the Ctrl. Canola fresh OM also significantly reduced RN/g root in Trials 1 and 3 compared with the Ctrl. In Trial 2, there were no significant differences among treatments for RN per root system (Fig. 2E) and only dry carinata had significantly lower RN/g root than the Ctrl (Fig. 2B). Reniform nematodes per gram of roots and per root system did not generally differ between crops with the fresh OM amendments and those with the dry OM amendments (Fig. 2).

#### Reniform nematode reproduction factor

**Figure 3:**
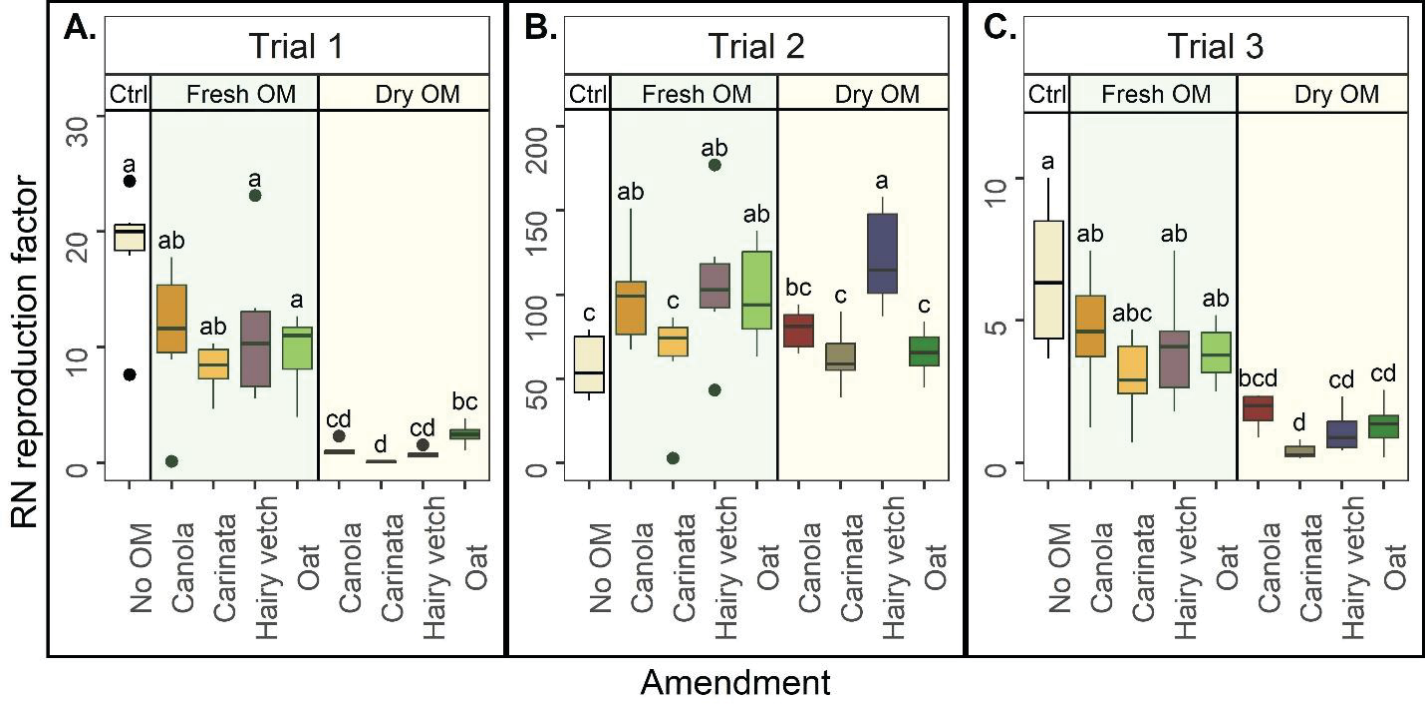
Reniform nematode (RN) reproduction factor (total RN per pot final population/inoculated initial population), by amendment, in Trial 1 (A), Trial 2 (B), and Trial 3 (C). Ctrl = control without organic matter (OM), Fresh OM = treatment with fresh OM, Dry OM = treatments with dry OM. Letters indicate treatment separation by Kruskal-Wallis with Bonferroni's method, *P* < 0.05. Data is displayed as boxplots.

**Figure 4:**
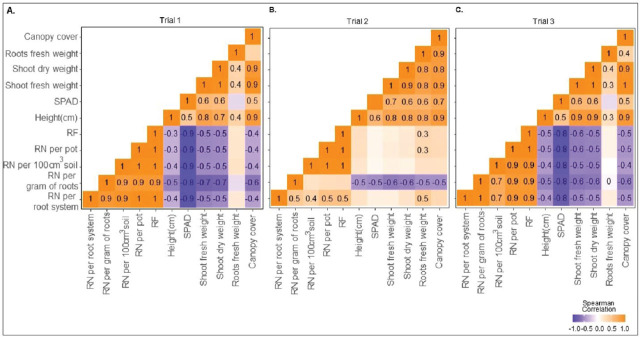
Spearman correlation between reniform nematode (RN) and plant parameters in Trial 1 (A), Trial 2 (B), and Trial 3 (C) in experiment 1. Numbers inside squares represent the rho value if *P* ≤ 0.05. Empty squares represent *P* ≥ 0.05. Signs before numbers and colors indicate the magnitude of the correlation. 1 (orange color) = perfect positive association of rank, −1 (blue color) = perfect negative association between ranks. RF = Reproduction factor.

The magnitude of RN reproduction and treatment effects on RN reproduction were different depending on the trial (Fig. 3). In Trials 1 and 3, all dry OM treatments decreased the RF compared to the Ctrl (Figs. 3A,C). In Trials 1 and 3, RF was generally lower for the dry OM amendments than the fresh OM amendments (Figs. 3A,C). In Trial 2, nevertheless, RF for all treatments were similar to or greater than the Ctrl (Fig. 3B). Again, there were few differences among crops within OM types. For Trials 1 and 3, RF did not vary among the fresh OM amendments (Fig. 3A). In Trial 2, however, carinata fresh OM had a lower RF than all other fresh OM amendments (Fig. 3B). Similarly, among the dry OM amendments, carinata had a lower RF than oat in Trial 1 and hairy vetch in Trial 2, respectively (Figs. 4A,B). In Trial 3, there were no differences in RF among the dry OM treatments (Fig. 3C).

#### Plant parameters

Height varied by trial depending on the treatment (Table 2). In general, cotton height was greater following dry amendments than in the Ctrl (inoculated with RN, but no OM) or the Ctrl− (no RN or OM) and was intermediate following fresh amendments. In Trial 1, cotton plants were taller following incorporation of dry OM of canola, hairy vetch, and oat compared to the Ctrl− or the Ctrl. The latter, in turn, had the shortest cotton. Fresh OM amendments were not different from the controls or the dry OM amendments. In Trial 2, dry OM, with the exception of oat, produced taller cotton plants than the Ctrl or Ctrl−. In Trial 3, cotton height following dry OM amendments was greater than the Ctrl, and the Ctrl− and did not vary among crops within dry amendment. The fresh OM amendments in Trial 3 had intermediate heights, greater than the Ctrl−, but not different from the Ctrl.

**Table 2.** Influence of winter crop organic matter incorporation on cotton growth and SPAD value in greenhouse conditions in the presence of reniform nematode.

	**Trial 1^a^**						
**Treatment**	**Canopy cover**	**Height (cm)**	**Roots fresh weight (g)**	**Shoot dry weight (g)**	**Shoot fresh weight (g)**	**SPAD**
*p*-value^b^	^***^	^***^	^***^	^***^	^***^	^***^
Control −^c^	6.1 ± 1.2 c	13.0 ± 1.2 bc	6.6 ± 0.9 b	2.1 ± 0.3 cd	5.2 ± 0.8 cd	43.0 ± 1.8 b
Control +^d^	5.7 ± 0.7 c	11.3 ± 0.8 c	9.2 ± 1.6 ab	1.5 ± 0.1 d	4.1 ± 0.4 d	34.0 ± 1.0 c
Fresh Amendment							
Canola	13.6 ± 0.9 abc	18.5 ± 0.9 abc	16.6 ± 3.4 a	3.4 ± 0.2 bc	9.2 ± 0.6 abc	42.2 ± 1.8 b
Carinata	9.1 ± 1.5 bc	16.3 ± 1.7 abc	10.6 ± 1.9 ab	2.0 ± 0.3 cd	5.9 ± 0.9 cd	45.4 ± 1.2 ab
Hairy vetch	11.5 ± 2.4 abc	15.4 ± 1.7 abc	7.4 ± 1 b	2.7 ± 0.4 bcd	7.3 ± 1.1 bcd	43.3 ± 1.1 b
Oat	8.4 ± 1.6 bc	17.7 ± 2.4 abc	7.5 ± 1.5 b	1.5 ± 0.2 d	4.5 ± 0.7 cd	43.4 ± 1.1 b
Dry Amendment							
Canola	19.2 ± 4.8 ab	21.5 ± 2.6 a	7.6 ± 1.4 b	3.8 ± 0.5 abc	11.7 ± 1.7 ab	49.4 ± 0.7 a
Carinata	15.7 ± 2.2 ab	20.6 ± 0.8 ab	8.0 ± 0.6 b	3.9 ± 0.4 ab	11.0 ± 1.1 ab	50.6 ± 0.3 a
Hairy vetch	19.0 ± 3.5 ab	21.5 ± 2.2 a	11.3 ± 2.0 ab	4.3 ± 0.5 ab	11.9 ± 1.4 ab	49.8 ± 0.5 a
Oat	21.4 ± 0.9 a	22.1 ± 0.7 a	8.8 ± 1.0 ab	5.3 ± 0.3 a	14.0 ± 1.1 a	49.5 ± 1.3 a

	**Trial 2^a^**						
**Treatment**	**Canopy cover**	**Height (cm)**	**Roots fresh weight (g)**	**Shoot dry weight (g)**	**Shoot fresh weight (g)**	**SPAD**
*p*-value^b^	^***^	^***^	^***^	^***^	^***^	^***^
Control −^c^	4.8 ± 0.6 b	13.9 ± 1.1 cd	6.7 ± 0.6 de	2.1 ± 0.2 ab	5.8 ± 0.5 abc	39.3 ± 4.5 ab
Control +^d^	1.7 ± 0.5 c	11.6 ± 1.5 d	3.4 ± 0.5 e	0.5 ± 0.1 c	1.8 ± 0.5 d	24.6 ± 2.2 c
Fresh Amendment						
Canola	8.7 ± 1.4 ab	16.6 ± 1.8 bcd	13.4 ± 1.2 abcd	2.1 ± 0.4 ab	7.8 ± 1.1 abc	33.0 ± 1.2 bc
Carinata	6.6 ± 0.9 b	16.6 ± 0.7 bcd	9.4 ± 0.7 bcd	1.9 ± 0.5 abc	5.8 ± 1.3 bc	38.0 ± 2.9 ab
Hairy vetch	5.4 ± 0.9 b	18.3 ± 0.7 abc	11.1 ± 1.5 abcd	1.5 ± 0.3 abc	5.1 ± 0.7 bc	33.7 ± 2.6 bc
Oat	5.4 ± 1.2 b	15.8 ± 1.6 bcd	9.1 ± 1.9 cd	1.6 ± 0.4 bc	4.0 ± 1.1 cd	32 ± 1.5 bc
Dry Amendment						
Canola	16.5 ± 3.1 a	23.1 ± 1.3 a	20.0 ± 1.9 a	3.5 ± 0.4 a	12 ± 1.4 a	47.3 ± 0.4 a
Carinata	10.6 ± 1.5 ab	19.3 ± 0.9 abc	18.0 ± 2.6 ab	2.6 ± 0.4 ab	8.7 ± 1.1 ab	46.5 ± 1.3 a
Hairy vetch	10.7 ± 0.6 ab	19.7 ± 0.4 ab	16.7 ± 1.7 abc	2.5 ± 0.3 ab	8.1 ± 0.9 ab	43.0 ± 0.7 ab
Oat	5.9 ± 1.6 b	14.7 ± 1.2 bcd	10.8 ± 2.8 bcd	1.4 ± 0.3 bc	5.3 ± 0.9 bc	35.3 ± 1.2 ab

	**Trial 3^a^**					
**Treatment**	**Canopy cover**	**Height (cm)**	**Roots fresh weight (g)**	**Shoot dry weight (g)**	**Shoot fresh weight (g)**	**SPAD**
*p*-value^b^	^***^	^***^	^***^	^***^	^***^	^***^
Control −^c^	9.3 ± 0.9 cd	8.0 ± 0.4 e	7.4 ± 0.3 b	1.9 ± 0.1 cd	6.1 ± 0.5 bc	50.9 ± 1.3 a
Control +^d^	5.1 ± 0.5 d	10.2 ± 0.3 d	4.4 ± 0.3 c	1.4 ± 0.1 d	4.3 ± 0.3 c	41.5 ± 1.4 b
Fresh Amendment						
Canola	9.7 ± 1.1 c	10.8 ± 0.6 cd	9.3 ± 0.7 ab	1.8 ± 0.1 cd	6.2 ± 0.4 bc	42.6 ± 0.9 b
Carinata	10.3 ± 1.3 abc	11.0 ± 0.9 cd	9.5 ± 1.2 ab	2.1 ± 0.2 bcd	6.8 ± 0.7 bc	47.0 ± 1.5 ab
Hairy vetch	10.2 ± 1.4 bc	10.6 ± 0.8 cd	11.0 ± 1.0 ab	1.9 ± 0.3 cd	6.3 ± 0.9 bc	42.9 ± 1.5 b
Oat	11.8 ± 1.5 abc	11.7 ± 1 bcd	11.5 ± 0.8 a	2.3 ± 0.2 abcd	7.5 ± 0.6 ab	43.6 ± 0.9 b
Dry Amendment						
Canola	15.2 ± 1.2 abc	12.6 ± 0.6 abc	8.1 ± 0.8 ab	2.7 ± 0.2 abc	9.5 ± 0.6 ab	47.4 ± 1.0 ab
Carinata	18.4 ± 2.5 a	14.7 ± 0.7 a	8.5 ± 0.9 ab	3.5 ± 0.4 a	11.3 ± 1.2 a	51.5 ± 0.8 a
Hairy vetch	17.1 ± 1.5 ab	13.8 ± 0.6 ab	7.6 ± 0.4 b	3.2 ± 0.1 ab	10.6 ± 0.6 a	51.5 ± 0.8 a
Oat	16.0 ± 2.2 abc	14.3 ± 0.6 a	8.8 ± 0.3 ab	2.9 ± 0.3 abc	9.4 ± 0.9 ab	49.5 ± 1.6 a

a Mean ± standard error. (*N* = 6)

b Letters within the same plant parameter and column indicate significant differences between treatments by trial (Tukey HSD, *P* ≤ 0.05).

“^***^”denotes ANOVA with *P* ≤ 0.01.

c Ctrl− indicates the treatment without nematodes and without OM

d Ctrl+ indicates the treatment with nematodes but without OM

The root fresh weight in Trial 1 was greater in cotton plants following canola fresh OM compared to the Ctrl−, the fresh OM from hairy vetch and oat, and the dry OM from canola and carinata (Table 2). In Trials 2 and 3, all treatments had greater root fresh weight than the Ctrl. In Trial 2, the dry OM amendments from canola, carinata, and oat produced greater cotton root fresh weight than the Ctrl−. In Trial 3, the fresh OM from oat had greater root weight than the Ctrland the hairy vetch dry OM amendment; there were no differences among the other treatments, which had intermediate root weight values.

In general, shoot dry weight of cotton was greater following dry amendments than the Ctrl, and intermediate or equal to the Ctrl following the fresh OM amendments (Table 2). In all trials, the dry weight of the cotton plants was greater than the Ctrl following the dry OM amendments, with the exception of dry OM from oat in Trial 2. The shoot dry weights of the cotton plants following the dry amendment from carinata and hairy vetch were also greater than the Ctrl− in Trials 1 and 3, but not in Trial 2. Cotton plants following fresh OM amendments were only different than the Ctrl after the canola amendment in Trial 1 and Trial 2, while none of the fresh OM amendments were different in the shoot dry weight than the Ctrl−. Overall, there were no consistent differences among crops within OM type.

Analogous to the other plant parameters evaluated, the shoot fresh weight was greater than with the Ctrl in the cotton plants after the dry OM amendments (Table 2). The shoot fresh weights after the fresh OM amendments were generally similar to the Ctrl and the Ctrl−. Similarly to previous plant parameters, there were no consistent differences in the shoot weight among crops within OM type.

The SPAD value was generally greater in the cotton plants after the dry OM amendments than with the fresh OM amendments (Table 2). The dry OM amendment from carinata, hairy vetch and oat had consistently greater SPAD value than the Ctrl in all the trials, and the dry OM amendment from canola had greater SPAD value than the Ctrl in Trials 1 and 2 but not in Trial 3. However, the dry OM amendments were only greater than the Ctrl− in Trial 1 and no different than Ctrl− in Trials 2 and 3. The fresh OM amendments had greater SPAD value than the Ctrl only in Trial 1 and the fresh OM from carinata in Trial 2. Nevertheless, the SPAD values of the cotton plants after the fresh OM amendments were not generally different than the Ctrl−, except in Trial 3, where canola, hairy vetch and oat had lower SPAD values than the Ctrl−. Additionally, no differences were detected in the SPAD value among crops among the dry OM or within the fresh OM treatments.

The canopy cover of the cotton plants after the dry OM amendments was consistently greater than the Ctrl in each trial (Table 2). The cotton canopy cover after the dry OM amendments was greater than the Ctrl− in Trial 1 and Trial 2 for canola, and Trial 1 and Trial 3 for carinata and hairy vetch. After the fresh OM amendments, the canopy cover from the cotton plants was greater than the Ctrl in Trials 2 and 3 but was not different than the Ctrl in Trial 1. Moreover, none of the fresh OM amendments had different canopy cover than the Ctrl− in the three trials. As had occurred with previous plant parameters, there were no differences among crops within the respective categories of those with dry or fresh OM treatments.

#### Spearman correlation

In Trials 1 and 3, the correlations between the RN parameters and plant parameters were negative, except for the root fresh weight, in which no correlation was seen with any of the nematode parameters (Figs. 4A,C). In these trials, the strongest magnitude of correlation was seen between the nematode parameters and the SPAD value. In Trial 2, only the abundance of RN per gram of roots was negatively correlated with all the plant parameters (Fig. 4B).

## Discussion

The results from this study suggest that under the same weight-weight ratio of dry or fresh OM amendments, dry amendments were effective at managing RN, while fresh amendments were not. In this research, the comparison between fresh and dry OM had practical implications, especially for a crop such as carinata that is left to dry in the field before incorporating into the soil. Previous reports suggest that in biofumigation, the amount of OM integrated into the soil is related to the efficacy of nematode management (Zasada and Ferris, 2004). In this experiment, fresh and dry amendments were used at the same weight of fresh or dry tissue, respectively, but the dry amendments were more concentrated because a larger volume of plant material was required to obtain the dry than the fresh amendments. Thus, the dry OM may have had higher concentrations of nematode-toxic compounds (Oka, 2010) than the fresh OM, leading to a greater suppression of RN by the dry OM treatments.

This comparison of equal amendment rates provided useful information for management decisions. However, given the results of this study, comparison of dry and fresh OM amendments derived from the same plant fresh weight would be a logical future research target, and could reflect a choice of incorporating plant residue from a prior crop when fresh or leaving it to dry. Additionally, in this study, plants were oven-dried. Therefore, there could be a variation in the response when the crop is left to naturally dry in the field. Thus, it would be useful to validate these results under field conditions.

In contrast to this study, where dry OM was more helpful in managing RN compared to the fresh OM amendments, a previous report determined no clear advantages to managing nematodes using dry residues compared to fresh residues of brassicaceous crops at 2% w/w, but saw a reduction of *M. incognita* galling in tomato with both types of OM (Stapleton and Duncan, 1998). The differing results could be related to the nematode and crop species used in the respective studies.

However, similar to this study, other studies have found dry OM to be effective for managing nematodes. For example, the incorporation of dry *Brassica* sp. at 2% w/w efficiently reduced the abundance of *Pratylenchus neglectus* (Potter et al., 1998). Additionally, the limited effect of fresh amendments on nematode populations has been previously reported. For example, green manure from carinata or canola leaves resulted in poor or no reduction of *Globodera pallida* (Lord et al., 2011), fresh OM from *B. oleraceae* var. *italica* at 2% w/w did not affect the ability of *M. javanica* to infect watermelon plants (Ploeg and Stapleton, 2001), and the incorporation of canola fresh OM at 1.3 to 2.7% w/w did not impact the abundances of *Mesocriconema ornatum*, *M. incognita,* and *M. javanica* (Johnson et al., 1992). Therefore, based on the results of this and previous research, fresh amendments are ineffective or inconsistent in managing nematodes, while dry OM is generally effective when applied at 2% w/w.

The poor effect of fresh amendments had been attributed to multiple factors, such as crop biomass production, insufficient disruption of the vegetative tissue, rapid degradation by microorganisms, or inappropriate soil conditions during incorporation (Dutta, 2019). All these considerations, however, do not exclusively apply to fresh OM, but to any biofumigant management strategy. One limitation of this study is that only a single amendment rate (2% w/w) was tested that was based on effective rates in other studies. This was useful for initially evaluating if dry or fresh residue from carinata or other crops affects RN, but more work would be needed to determine if crop residues are effective in production conditions. Depending on costs and biomass production, the 2% w/w rate tested in this study may not be feasible in field conditions. A future study using various amendment rates, such as biomass from crops grown at field planting density—as previously discussed—could help better elucidate the practical efficacy of winter crop amendments for managing RN.

The crop was not a determinant factor in the reduction of RN. In general, crops did not differ in their effects on the RN reproduction and abundance. According to previous research, biofumigation is not exclusively related to the concentration of the toxic compound, but to the efficiency of its content (Motisi et al., 2010). This suggests that the combination of volume and efficiency of nematotoxic compounds produced by the crops tested were similar, but this was not quantified. Another possible explanation for the lack of differences among crops in this study is that physical (such as porosity, texture, aeration, water holding capacity) and chemical (such as pH, electric conductivity, enzymes) soil properties changed according to OM type and consequently helped manage RN (Widmer et al., 2002) and impacted crop development. In this study, organic matter was uniformly incorporated into the soil and the size of the OM particles was the same within each OM type, so any physical changes to the soil were probably similar across crops. Again, these properties were not measured in this study, and future research would be needed to determine the mechanism for RN management by OM amendment with these specific crops.

In contrast to our results, in previous studies, brassicaceous crops resulted in greater suppression of nematodes compared to other non-brassicaceous crops. For instance, canola (cultivar Jupiter) resulted in a greater reduction of *M. chitwoodi* abundance compared to wheat (*Triticum aestivum*), a graminaceous crop (Mojtahedi et al., 1991), and leachates from canola also caused less suppression of RN compared to the fabaceous crop *Crotalaria juncea* (Wang et al., 2001). Another study reported differences in nematode suppression among amendments of different brassicaceous crops. *B. juncea*, a plant with higher glucosinolate content, provided greater suppression of *Tylenchulus semipenetrans* compared to *B. oleraceae* var. *botrytis*, a crop with lower glucosinolate content (Zasada and Ferris, 2004). However, in this study, no differences were seen between carinata and canola in RN abundance from other factors or in reproduction, although carinata has greater glucosinolate content than other brassicas, such as canola (Rahman et al., 2018; Hagos et al., 2020).

In Trial 2, half of the OM treatments had similar reproduction of RN compared to the control without OM, and half had greater reproduction. This was despite dry OM treatments being effective in the other trials. Similarly, inconsistent results in plant-parasitic nematode management have been reported when organic matter amendments have been used (McSorley, 2011). The temperature during winter crop and cotton growth could be one of the factors causing these inconsistencies.

External conditions can affect secondary metabolite production in plants. For instance, glucosinolate production can be affected by temperature, cultivation time, and relative humidity (Shim et al., 2018). In Trial 2, the maximum temperature for the winter crops during growth was higher than in Trials 1 and 3, and this probably affected the growth of the winter crops, causing alterations in the production of the nematicidal compounds. The average temperature for the cotton during growing was also greater during Trial 2 than in Trials 1 and 3, which increases RN reproduction; this was reflected in the relatively greater RF in Trial 2 (approximately 50–125) than in Trials 1 and 3 (less than 20 and 10, respectively) (Rebois, 1973).

Nematode population increase after OM incorporation has been previously reported (Thoden et al., 2011). For example, *P. penetrans* abundance was higher in potato soils treated with compost or manure compared to untreated soils (Kimpinski et al., 2003). The isothiocyanates levels of a plant can also be influenced by the soil pH, temperature, and moisture (Andréasson et al., 2001; del Carmen Martínez-Ballesta et al., 2013). Therefore, practical application of OM in the field should be done while carefully considering the external conditions and factors to avoid the increase of RN population. Under the high RN-pressure conditions of Trial 2, the carinata amendment held up the best, as it was the only crop that maintained similar RN reproduction to the Ctrl for both dry and fresh amendments.

In this study, soil samples with nematodes and amendments were left in the greenhouse for one week before planting cotton to avoid phytotoxicity during cotton germination and to simulate field management practices. Zhou and Everts (2004) reported that amendments from plant residues could produce phytotoxicity, especially if the subsequent crop is planted a short period after OM incorporation (Stapleton, 2006). For example, oat contains compounds that can inhibit the radical elongation of some plants (Grimmer and Masiunas, 2005); hairy vetch can prevent seed germination and has even been used to reduce the persistence of some weeds (Mohler et al., 2018); and brassicas are also known for their phytotoxic effects and the potential of their amendments to inhibit some plants (Haramoto and Gallandt, 2004).

In this study, treatments both without amendment and without RN (Ctrl−) were included when analyzing the plant parameters to compare the growth and physiology of the cotton plants with and without RN. That treatment (Ctrl−) was not included when analyzing the nematode parameters because it did not include nematodes. Treatment without amendment but with RN (Ctrl) was included to compare the treatments with OM on the RN population and the growth and physiology of the cotton plants. In this experiment, as growth parameters (height, root fresh weight, shoot dry and fresh weight, and canopy cover) did not generally differ between the treatments without nematodes and OM (Ctrl−) and with nematodes but without OM (Ctrl); thus, reniform nematodes by themselves did not impair cotton growth.

This contradicts previous reports of RN causing fresh weight reduction in 4-month cotton plants in the greenhouse (Jones et al., 1959). Although there was a numerical trend in lower cotton growth in several plant parameters (height, shoot dry and fresh weight, canopy cover) in the Ctrl compared to the Ctrl−, it is possible that as plants were not left to mature in this study, the effect of the nematodes on their growth was not enough to reflect differences in the growth parameters evaluated between the Ctrl and the Ctrl−. As previously noted, RN is known to be damaging to cotton grown under field conditions (Dyer et al., 2020).

The SPAD value, nevertheless, was consistently greater in the Ctrl− than in the Ctrl. SPAD value is a physiological indirect measure of leaf chlorophyll content (Monostori et al., 2016) and has been demonstrated to be an efficient method to determine the concentration of photosynthetic pigments in cotton leaves (Brito et al., 2011) and has been found to be an effective cotton yield predictor (Zhou and Yin, 2018). The chlorophyll content is one of the most significant aspects in influencing photosynthetic efficiency (Mao et al., 2007). In this research in the presence of nematodes, SPAD value was the plant variable most consistently correlated with the abundance of nematodes. This correlation was negative, meaning when nematode abundances were greater the leaf chlorophyll content was lower. In a plant affected by nematodes, nutrients can move from the host to the pathogen, affecting the carbon transport and metabolism of the host and negatively altering the photosynthetic capacity of the plant (Breia et al., 2021). Therefore, the results from this research suggest that nematodes produce a negative effect on the photosynthetic capacity of cotton plants that could later translate to reduced yield.

In general, there were no differences in the plant growth or SPAD values between dry or fresh amendments of the same crop. Additionally, fresh OM provided no observed benefit for cotton growth or SPAD values compared to the treatment without nematodes or any OM at all. However, OM amendments in general could provide some benefit in the plant growth parameters or SPAD values. For example, the root fresh weight and canopy cover were greater in two out of three trials with any of the fresh OM amendments, the shoot dry weight was greater with canola OM in two out of three trials, and carinata, hairy vetch, and oat provided a benefit in the SPAD compared to the treatment with nematodes but without OM (Ctrl). Likewise, in presence of nematodes, the incorporation of dry OM from canola, hairy vetch, or carinata could provide growth effects in cotton in all the plant parameters evaluated, and greater SPAD values than the treatment without OM (Ctrl). These responses could be because organic matter releases nutrients, supports soil structure, and increases ion exchange (D’Addabbo, 1995). These factors promote root and plant growth, making the plant more resilient to nematodes (Akhtar and Alam, 1993). Consistent with our study, previous research reported that OM can be beneficial in cotton growth. For instance, barley residue soil incorporation increased the plant biomass, nutrient uptake, leaf area, seed cotton yield, and photosynthesis of cotton plants (Lv et al., 2021).

In conclusion, the overall results suggest that dry OM are better than fresh OM amendments, under the same OM weight-weight ratio, for managing RN. This implies that OM concentration could have a substantial role in successfully managing RN. Under these conditions, crop type was not a determining factor in managing RN. Considering the practical management implications, dry OM from carinata, canola, or oat could be beneficial for managing RN. Photosynthesis of cotton plants was also found to be affected by RN, and SPAD values could help to indicate nematode presence in the plants. Under the application of the OM amendments, crop growth was variable and did not always relate to nematode abundances. Future research is needed to more fully assess the efficacy of carinata and other winter crops at managing RN. Specific directions for future research include field studies, assessment of OM amendment in combination with rotation, and further OM application rate studies.
